# Metformin and erlotinib synergize to inhibit basal breast cancer

**DOI:** 10.18632/oncotarget.2391

**Published:** 2014-11-04

**Authors:** Ying-Ka Ingar Lau, Xing Du, Vinayak Reyannavar, Benjamin Hopkins, Jacquelyn Shaw, Eliana Bessler, Tiffany Thomas, Maira M. Pires, Megan Keniry, Ramon E. Parsons, Serge Cremers, Matthias Szabolcs, Matthew A. Maurer

**Affiliations:** ^1^ Herbert Irving Comprehensive Cancer Center, Columbia University, New York, NY 10032; ^2^ Department of Medicine, Columbia University, New York, NY 10032; ^3^ Icahn School of Medicine at Mount Sinai, New York, NY 10029; ^4^ Current address, Albert Einstein College of Medicine, Bronx, NY 10461; ^5^ Irving Institute for Clinical and Translational Research, Columbia University, New York, NY 10032; ^6^ Current address, Biology Department, University of Texas Pan American, Edinburg, TX 78539; ^7^ Department of Pathology, Herbert Irving Comprehensive Cancer Center, Columbia University, New York, NY 10032

**Keywords:** breast cancer, metformin, erlotinib, PTEN, EGFR

## Abstract

Basal-like breast cancers (BBCs) are enriched for increased EGFR expression and decreased expression of PTEN. We found that treatment with metformin and erlotinib synergistically induced apoptosis in a subset of BBC cell lines. The drug combination led to enhanced reduction of EGFR, AKT, S6 and 4EBP1 phosphorylation, as well as prevented colony formation and inhibited mammosphere outgrowth. Our data with other compounds suggested that biguanides combined with EGFR inhibitors have the potential to outperform other targeted drug combinations and could be employed in other breast cancer subtypes, as well as other tumor types, with activated EGFR and PI3K signaling. Analysis of BBC cell line alterations led to the hypothesis that loss of PTEN sensitized cells to the drug combination which was confirmed using isogenic cell line models with and without PTEN expression. Combined metformin and erlotinib led to partial regression of PTEN-null and EGFR-amplified xenografted MDA-MB-468 BBC tumors with evidence of significant apoptosis, reduction of EGFR and AKT signaling, and lack of altered plasma insulin levels. Combined treatment also inhibited xenografted PTEN null HCC-70 BBC cells. Measurement of trough plasma drug levels in xenografted mice and a separately performed pharmacokinetics modeling study support possible clinical translation.

## INTRODUCTION

Approximately 80% of breast cancers classified by gene expression as basal-like are identified clinically as triple negative [1]. Triple negative breast cancers (TNBCs) are defined by a lack of estrogen receptor expression and progesterone receptor expression, as well as an absence of human epidermal growth factor receptor 2 (*ERBB2/HER2*) amplification. BBCs are heterogeneous, but common molecular aberrations include overexpression of epidermal growth factor receptor (EGFR), loss of expression of phosphatase and tensin homolog deleted on chromosome ten (PTEN), as well as mutation of *BRCA1*, *PIK3CA* and *p53* [2–5]. More recently, it has been shown that many BBCs harbor decreased expression of the PTPN12 tyrosine phosphatase and inositol polyphosphate 4-phosphatase type II (INPP4B), leading respectively to increased growth factor and PI3K pathway activation [6–8]. Overall, BBCs have the highest PI3K/AKT pathway activity among the different breast cancer subtypes [1]. While we await clinical exploitation of these findings, BBCs retain a high rate of recurrence and death [9].

EGFR, due to its oncogenic properties and its overexpression in BBCs, provides an opportunity for targeted therapies [10]. Recently published data from the work of The Cancer Genome Atlas (TCGA) indicates that approximately 23% of BBCs harbor *EGFR* gene copy number gain [1]. Although EGFR expression correlates with poor prognosis, clinical trials incorporating EGFR inhibitors in TNBC have yielded only modest clinical results [11]. This may be due to the heterogeneous nature of BBCs in which not only the expression of EGFR is variable but also the activity of EGFR and dependence of the tumor on that activity. Furthermore, plausible explanations for primary resistance to single agent EGFR targeted therapy include continued activation of alternate receptors tyrosine kinases [e.g. c-Met and insulin-like growth factor 1 receptor (IGF1R)], signal feedback, or de-coupling EGFR from downstream AKT signaling through loss of PTEN or INPP4B [7, 12–15]. Therefore, rational drug combinations with the goal of potentiating the effect of EGFR inhibitors in BBCs should be explored.

Metformin, a type 2 diabetes drug, has demonstrated antitumor effects in multiple cancer models [16–18]. Metformin has been shown to reduce EGFR, mitogen-activated protein kinase (MAPK) and AKT signaling in breast cancer cell lines, and selectively induced apoptosis in TNBC cells [19, 20]. However, the clinical relevance of these *in vitro* findings remains uncertain due to the high doses of metformin required. Recently, metformin was shown to selectively kill tumor initiating cells at doses as low as 100 μM with corresponding potentiation of chemotherapy efficacy in xenograft models [21]. In addition, epidemiologic studies show that diabetic patients taking metformin have a lower mortality rate as well as a decreased risk of developing breast cancer [22, 23], although these results remain debatable due to possible methodology shortcomings [24, 25]. To clarify such ambiguity, continued research into the potential use of metformin as a cancer therapeutic is worthwhile.

The mechanisms of metformin's anti-neoplastic properties are controversial. Metformin can reduce circulating glucose and insulin levels by inhibiting gluconeogenesis in the liver. This is accomplished through metformin's ability to indirectly activate AMP-activated protein kinase (AMPK) by inhibiting oxidative phosphorylation in cells [26]. In cancer cells, this inhibition leads to reduced ATP production and cellular energy crisis[27]. Among its many substrates, activated AMPK inhibits mammalian target of rapamycin complex-1 (mTORC1) output and lipogenesis by phosphorylating tuberous sclerosis complex 2 (TSC2) and acetyl-CoA-carboxylase (ACC), respectively [28, 29]. In addition, metformin has recently been shown to directly inhibit mTORC1 activity in a RAG GTPase dependent manner and indirectly through the p53-REDD1 axis [30, 31]. Metformin has been shown to inhibit tumors in mice both by decreasing circulating growth factors and by directly inhibiting tumor cell growth and survival pathways. In one study, metformin delayed the outgrowth of tumors in *PTEN* heterozygous mice without altering the plasma insulin or IGF-1 levels [32]. In contrast, metformin prevented tumor formation in a toxin-mediated mouse model of lung cancer while reducing circulating IGF-1 and insulin levels, but without demonstrated ability to activate AMPK in lung tissue [18]. Although these data have not led to a unified mechanism of how metformin could inhibit or prevent human cancers, epidemiologic and preclinical evidence has inspired ongoing clinical trials.

Metformin's ability to activate AMPK, as well as deactivate EGFR, AKT and mTORC1 provides a rationale for assessing whether metformin can synergize with EGFR inhibition to treat BBCs. In this study, we demonstrated that erlotinib (an EGFR kinase inhibitor) and metformin synergistically inhibited the growth and induced cell death in a subset of BBC cell lines with accompanying enhanced reduction of EGFR, AKT and mTORC1 signaling. We showed in an isogenic MCF10A cell model that loss of PTEN sensitized cells to the drug combination and conversely, introducing PTEN into PTEN-null tumor cell lines desensitized cells to the drug combination. This synergy was confirmed *in vivo* by demonstrating significant reduction in tumor burden after combined drug treatment as compared to treatment with either drug alone.

## RESULTS

### A subset of BBC lines is sensitive to combined treatment with metformin and erlotinib

**Figure 1:**
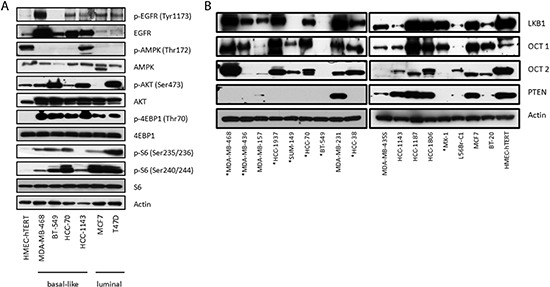
Dysregulation of signaling pathways and loss of PTEN expression in a panel of breast cancer cell lines **(A)** Immunoblots of members of the EGFR, PI3K, mTOR, and AMPK signaling pathways in proliferating breast cancer cell lines **(B)** Immunoblots assessing the levels of PTEN, LKB1 and the cation transporters OCT1 and OCT2. *PTEN* mutant lines are marked with (*).

To investigate the cell signaling status of various direct and indirect targets of erlotinib and metformin in BBCs, we examined the phosphorylation levels of EGFR, AMPK, AKT, S6 and 4E-binding protein 1 (4EBP1) in a panel of breast cancer cell lines. In general, there is increased phosphorylation of EGFR, AKT, S6 and 4EBP1 in breast cancer cell lines compared with immortalized non-transformed human mammary epithelial cells (HMEC-hTERT), as well as a significant decrease in AMPK phosphorylation at Thr172 (Fig. 1A). Although the luminal breast cancer lines MCF7 and T47D expressed very low levels of total EGFR, they exhibited significant EGFR phosphorylation. The consistent activation of EGFR and the phosphoinositide 3-kinase (PI3K) pathway across various BBC lines, as well as a decrease in AMPK signal, provided a strong rationale for testing combined treatment with erlotinib and metformin.

To investigate whether combined metformin and erlotinib treatment is effective in BBCs, a panel of 17 breast cancer cell lines (16 BBC lines and one luminal line) was screened for synergy over a range of metformin (0.5 to 4 mM) and erlotinib (2 to 4 μM) doses. We found that the drug combination is synergistic in 8 of the lines (47.1%), additive in 4 (23.5%) and antagonistic in 5 others (29.4%) (Table 1). To test that the observed synergy was not due to off target effects of erlotinib, we repeated the experiment using BIBW-2992 (a dual EGFR/HER2 tyrosine kinase inhibitor) and metformin in HER2-negative MDA-MB-468 cells and obtained similar results (Suppl. Table 1). These results demonstrate that inhibition of EGFR combined with the effects of metformin can synergistically inhibit the growth of a subset of BBC cell lines.

**Table 1:** Combination index of metformin and erlotinib treatment

Synergistic (CI)	Additive (CI)	Antagonistic (CI)
MDA-MB-468* (0.44)	MX-1* (1.19)	HCC-1187 (1.79)
MCF7 (0.46)	HCC-1143 (0.97)	HCC-38* (49.8)
MDA-MB-435S (0.27)	BT-549* (1.00)	MDA-MB-157 (2.04)
BT-20 (0.56)	SUM-149* (0.82)	MDA-MB-231 (15.4)
HCC-1937* (0.61)		HCC-1806 (1.25)
HCC-70* (0.21)		
L56Br-C1 (0.57)		
MDA-MB-436* (0.36)		

17 cell lines were treated with metformin and erlotinib as well as their combinations at various doses (6 replicates per treatment) and combination indices (CI) at metformin dose of 2 mM and erlotinib dose of 4 μM were determined using the software Compusyn. *PTEN* mutant lines are marked with (*)

To probe what may contribute to the combined drug synergy, we assessed the expression of OCT1, OCT2, liver kinase beta 1 (LKB1) and PTEN in the BBC lines. The OCTs are cation transporters of metformin and their expression varies in different tissues [33–36]. We found that the majority of cell lines, including HMEC-hTERT, expressed significant amounts of both OCT1 and OCT2, indicating that differential sensitivity to the drug combination was not due to limitations in metformin transport (Fig. 1B). BT-549, with almost no expression of either OCT protein, is one of the lines in the “additive group” which is consistent with the existence of known additional metformin transporters (e.g. OCT3) [33]. In addition, analysis of RNA-seq results of the TCGA BBC data with z-score threshold of ±1 using the cBio Cancer Genomics Portal [37] showed that a significant number of samples up-regulate gene expression of these OCT proteins, including OCT3 [[1]; data not shown]. This suggests that metformin can effectively enter breast cancer cells. While we found reduced LKB1 expression (upstream of AMPK) in multiple cell lines, only SUM-149 cells completely lacked LKB1 expression. We also noted that all the lines with known *PTEN* mutations fall into either the synergistic or additive category with the exception of the HCC-38 cell line, which upon review of the Sanger database contains an interchromosomal breakpoint within *PTEN* (Table 1), and confirmed that all the *PTEN* mutant lines lack PTEN expression (Fig. 1B). Therefore *PTEN* mutation was noted as a possible determinant of sensitivity to combined treatment.

### The combination of metformin and erlotinib inhibits EGFR, AKT, S6 and 4EBP1 signaling

**Figure 2:**
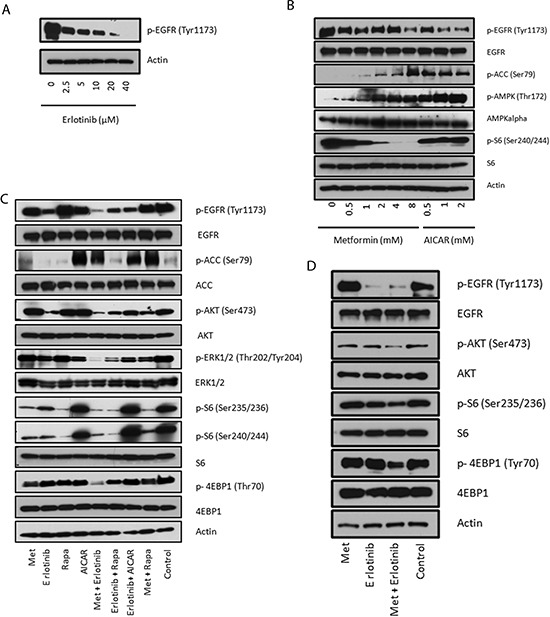
Combined treatment with metformin and erlotinib synergistically reduces EGFR, AKT, S6 and 4EBP signaling **(A)** Immunoblot of MDA-MB-468 cells treated for 24 hours with the indicated concentrations of erlotinib. **(B)** Immunoblots evaluating the dose-dependent signaling changes in EGFR, ACC, AMPK and S6 under metformin and AICAR treatments. **(C)** Immunoblots of signaling changes of EGFR, AKT, Erk1/2, S6 and 4EBP1 in MDA-MB-468 cells treated with metformin (2 mM), erlotinib (4 μM), rapamycin (10 nM) and AICAR (2 mM) and their combinations for 24 hours. **(D)** Immunoblots of signaling changes of AKT, S6 and 4EBP1 in BT-20 cells treated with metformin (2 mM), erlotinib (4 μM) and their combination for 24 hours.

MDA-MB-468 cells, highly sensitive to the drug combination, have known mutational abrogation of *PTEN* and *p53*, as well as amplification of *EGFR*. MDA-MB-468 cells therefore serve as a good model for TNBC with highly active EGFR signaling. MDA-MB-468 cells showed dose-dependent response (reduction of EGFR signaling) to erlotinib ranging from 2.5 to 40 μM but doses required to significantly inhibit EGFR phosphorylation are higher than what is achievable in patients treated with approved doses and schedules (Fig. 2A). According to the erlotinib product information (Roche), the median minimum and maximum steady state plasma levels of erlotinib, in patients treated at a dose of 150 mg daily, are 2.7 μM and 4.6 μM respectively. The incomplete blockade of EGFR signaling by erlotinib *in vitro* at doses corresponding to median clinically achievable plasma levels highlights a need to identify a synergistic combination. We then investigated the effect of metformin on MDA-MB-468 cells and compared metformin's effects with those of another AMPK agonist, 5-aminoimidazole-4-carboxamide riboside (AICAR). As expected, there was a dose-dependent phosphorylation of AMPK and ACC, a downstream substrate of AMPK, when treated with metformin or AICAR (Fig. 2B). However, there was a strong dose-dependent inhibitory effect on the phosphorylation of S6 using metformin that was not observed when cells were treated with AICAR, indicating this inhibitory effect on S6 in MDA-MB-468 cells was AMPK independent (Fig. 2B).

To test whether metformin and erlotinib could cooperate in sustained inhibition of relevant signaling pathways, we analyzed cell signaling changes 24 hours after various drug treatments (metformin, erlotinib, AICAR and rapamycin) in MDA-MB-468 cells. We included AICAR and rapamycin since metformin has been shown to directly or indirectly act on their targets. We observed potentiation of erlotinib-induced inhibition of EGFR, ERK1/2 and AKT signaling with the addition of metformin which was not observed in the other tested drug combinations: erlotinib and rapamycin, erlotinib and AICAR, or metformin and rapamycin (Fig. 2C). Although rapamycin is effective at inhibiting the mTORC1 S6 output, it is a poor inhibitor of 4EBP1 phosphorylation (Fig. 2C). Metformin, on the other hand, decreased phosphorylation of both S6 and 4EBP1 when used alone, and both outputs were further inhibited when metformin was combined with erlotinib. AICAR treatment lacked these effects, again indicating that the enhanced inhibition on cell signaling with metformin observed is not solely AMPK-dependent (Fig. 2C). We confirmed the same potentiation of inhibitory effects on AKT, S6 and 4EBP1 signaling in another BBC cell line, BT-20, which also has a strong basal EGFR signal output (Fig. 2D). We used an RTK antibody array to simultaneously assess whether other signaling pathways are changed by combination treatment, and found the levels of phosphorylated RTKs such as HER2, HER3, IGF-IR, and InsR were generally weak in MDA-MB-468 cells, and no significant changes were found (Suppl. Fig. 1A).

### Metformin and erlotinib combined to induce apoptosis, reduce clonogenicity, and inhibit mammosphere formation

**Figure 3:**
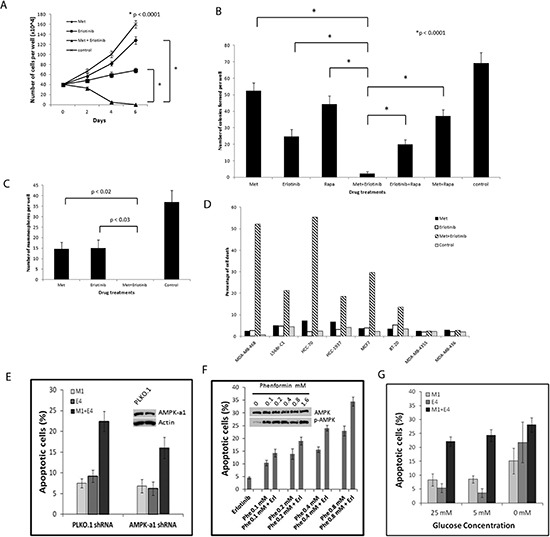
Combined treatment with metformin and erlotinib induces cell death in various breast cancer cell lines and reduces clonogenicity as well as mammospheres formation in MDA-MB-468 cells **(A)** Proliferation assay of MDA-MB-468 cells treated with metformin (1 mM), erlotinib (4 μM), and their combination over 6 days. **(B)** Clonogenic assay on MDA-MB-468 cells treated with metformin (0.5 mM), erlotinib (4 μM), rapamycin (10 nM) and their combination over 2 weeks. **(C)** Mammosphere formation assay on MDA-MB-468 cells treated with metformin (2 mM), erlotinib (4 μM) and their combination for 6 days. **(D)** 8 cell lines were treated with metformin (0.5, 1 or 2 mM) and erlotinib (4 μM) and their combination for 3 days and assayed for cell death using the FITC Annexin V Apoptosis Detection Kit I for flow cytometry analysis. This data is representative of the results from 3 separate experiments for each cell line. **(E)**, MDA-MB-468 cells were transduced with scramble PLKO.1 and AMPK-a1 shRNA lentiviruses and subjected to puromycin selection. Cells were further treated with vehicle control, 1 mM metformin (M1), 4 uM erlotinib (E4), or combination (M1+E4). Apoptotic cells were detected with Annexin V-FITC and PI staining. Apoptotic cell percentage from vehicle control was deducted from treatment groups. Mean +− S.D. of Annexin-V positive cells were shown (n=4). **(F)**, MDA-MB-468 cells were treated with 4uM erlotinib and different doses of phenformin. Mean +− S.D. of Annexin-V positive cells were shown (n=3). To detect the level of p-AMPK, cells were treated with phenformin for 24 hr. **(G)**, MDA-MB-468 cells were treated with vehicle control, 1 mM metformin (M1), 4 uM erlotinib (E4), or combination (M1+E4) in growth medium (containing 10% FBS, 4 mM glutamine) supplemented with 25 mM, 5 mM or 0 mM glucose. Mean +− S.D. of Annexin-V positive cells were shown (n=3).

Assessment of cell proliferation and apoptosis in MDA-MB-468 cells under different drug treatments showed that the combination of metformin and erlotinib induced cell death, while each single agent treatment only partially inhibited proliferation and was unable to induce apoptosis (Fig. 3A). Western blot analysis showed significant increased expression of cleaved-caspase 3 for cells under combination treatment as compared with single agent treatments (Suppl. Fig. 1B). We also performed a cytotoxic clonogenic assay in MDA-MB-468 cells and observed that the combination treatment of metformin and erlotinib significantly reduced, and almost eliminated, colony formation as compared with single agent treatments (Fig. 3B). We included rapamycin for comparison and observed that the metformin and erlotinib combination was superior to the rapamycin and erlotinib combination at a dosage that resulted in similar S6 inhibition. We also compared the effect of metformin and erlotinib versus their combinations with other inhibitors of relevant pathways revealed in our signaling experiment in MDA-MB-468 cells. These combinations include combining metformin with MK2206 (an allosteric AKT inhibitor), metformin with GSK1120212 (an allosteric MEK1 and MEK2 inhibitor), and erlotinib with AZD8055 (an ATP-competitive mTOR kinase inhibitor). We found that when these drugs were all used at their IC40 concentrations, the combination of metformin with erlotinib was superior to all other combinations tested as measured by growth inhibition over 6 days in MDA-MB-468 cells (Suppl. Fig. 2).

To investigate the effect of the drug combination on an enriched cancer stem cell population that forms mammospheres in non-attaching and serum-free culture conditions, we performed mammosphere formation assays in the presence of different drug treatments. We observed complete inhibition of mammosphere formation in MDA-MB-468 cells treated with both drugs while treatment with metformin or erlotinib as single agents only partially inhibited mammosphere formation (Fig. 3C). The ability of the combination to inhibit mammosphere outgrowth was confirmed in other BBC cell lines and suggested a more indiscriminant effect since the inhibition was seen in lines that showed only additive, or even antagonistic effects in our cell viability assays (Suppl. Fig. 3A-B).

To further confirm the ability of the drug combination of metformin and erlotinib to induce apoptosis, we tested the eight cell lines that were synergistically inhibited by the drug combination in the cell viability assay (Table 1) using flow cytometry to assay for cell death. Six of the eight cell lines (MDA-MB-468, HCC-1937, L56BrC1, HCC70, MCF7 and BT-20) exhibited significant cell death, ranging from 13.6% to 55.4%, under the combination treatment as compared with no increase in cell death with single drug treatments (Fig. 3D).

To further explore whether AMPK is involved in the synergistic effect from the combination treatment, we knocked down AMPK-a1 using lentiviral shRNA. Although AMPK-a1 knockdown generally made the MDA-MB-468 cells more resistant to metformin (Suppl. Fig. 4), the combination of metformin and erlotinib still produced synergistic cell death (Fig. 3E). We also tried a more potent biguanide phenformin. 0.1 mM of phenformin induced strong AMPK phosphorylation comparable to 0.8 mM, but the synergistic effect from combination with erlotinib was only evident at higher doses as 0.4 or 0.8 mM (Fig. 3F). These data along with the data from AICAR (Fig. 2C) suggest that AMPK activation is not solely responsible for the synergistic effect for the combination of metformin or phenformin with erlotinib.

Recent studies show that carbon sources affect the sensitivity to metformin [27, 38, 39]. We tested the effect of glucose on the synergism of metformin and erlotinib combination. 25 mM is the high glucose concentration in regular DMEM medium for MDA-MB-468, and 5 mM of glucose is close to physiological level found in serum. We found that the synergistic effect at 5 mM was comparable to 25 mM (Fig. 3G), but diminished in culture medium without glucose, although cells were generally more sensitive to either drug alone or combination under extreme low glucose condition.

Consistent with endogenous BIM expression in cancer cells being a determinant of apoptotic responsiveness to kinase inhibitors, including EGFR inhibitors [40], we observed that the two lines that did not show evidence of apoptosis upon combined drug traetment (MDA-MB-436 and MDA-MB-435S) expressed the lowest amount of endogenous BIM protein (Suppl. Fig. 5). All together, these results show that metformin combined with erlotinib synergize to inhibit a subset of breast cancer cell lines grown and assayed in multiple *in vitro* contexts.

### PTEN is one determinant of sensitivity to combined treatment with metformin and erlotinib

**Figure 4:**
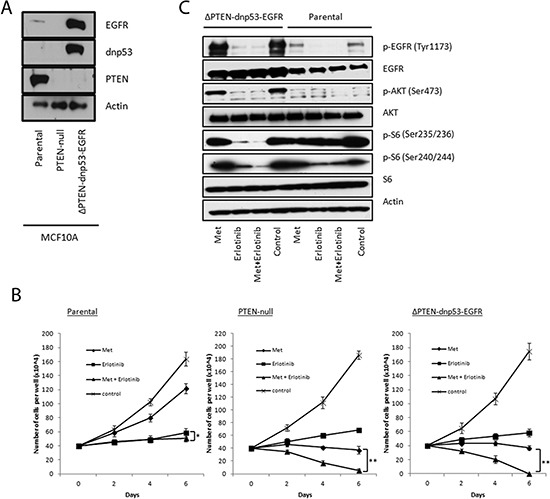
PTEN-null isogenic MCF10A cells shows increased sensitivity towards combined treatment with metformin and erlotinib **(A)** Immunoblots confirming genetic alterations in the MCF10A isogenic lines. **(B)** Proliferation assays of parental, PTEN-null and ΔPTEN-dnp53-EGFR MCF10A cells treated with metformin (4 mM), erlotinib (4 μM) and their combinaton over 6 days. 6 replicates were used per treatment (* represents p < 0.03 and ** represents p < 0.0001). **(C)** Immunoblot analysis of EGFR, AKT and S6 signaling in parental and ΔPTEN-dnp53-EGFR MCF10A cells treated with metformin (4 mM), erlotinib (4 μM) and their combination for 1 hour.

To further investigate whether PTEN affects sensitivity towards the drug combination, we utilized isogenic cell line models. We first compared a PTEN-null MCF10A line, derived by deletion of exon 2 of *PTEN* on both alleles, with its parental MCF10A line. In addition, we compared the MCF10A parental line with the PTEN-null line engineered to overexpress both dominant negative p53 (dnp53) and wild type EGFR (named “ΔPTEN-dnp53-EGFR”) [41], mimicking these common concurrent genetic alterations present in BBCs (Fig. 4A). We observed that the combination treatment induced cell death in the PTEN-null isogenic cells and MCF10A ΔPTEN-dnp53-EGFR cells while only inhibiting proliferation in the PTEN wild type MCF10A parental cells (Fig. 4B). Both PTEN-null cell lines were also more sensitive towards metformin treatment as compared with the parental line. We then looked for differential inhibition of signaling pathways between the cell lines. As expected, phosphorylation of EGFR and AKT were both significantly upregulated in the ΔPTEN-dnp53-EGFR line and substantially inhibited by treatment with erlotinib (Fig. 4C). Interestingly, the synergy of the combined treatment could be clearly observed through enhanced inhibition of S6 in ΔPTEN-dnp53-EGFR cells as compared with single agent treatments, and this effect was less pronounced in the MCF10A parental line. To assess the converse isogenic experiment, we introduced PTEN into the PTEN-null cell lines MDA-MB-468 and HCC-1937 which, in both cases, resulted in reduced AKT phosphorylation and a decrease in synergy (increased CI) to the drug combination (Suppl. Fig. 6A-D). These results indicate that loss of PTEN sensitizes breast cancer cells to combined metformin and erlotinib treatment and supports the assertion that PTEN status is one determinant of combined drug treatment sensitivity.

To test whether other PI3K pathway alterations can induce the synergism, and whether such synergism is applicable to other cancer types, we treated glioma cancer cell line H4, lung cancer cell line NCI-H1975, and ovarian cancer cell line CAOV-3 with metformin or phenformin (when cell line is resistant to metformin), and erlotinib. Based on cBio cancer genomics portal [37], H4 cell line has *PTEN* homozygous deletion and *EGFR* amplification; NCI-H1975 and CAOV-3 both have a *PIK3CA* mutation and an *EGFR* mutation. All three cell lines showed synergistic and/or additive cell death under combination treatment (Suppl. Fig. 7), which further confirms that PTEN status and/or PI3K pathway alteration may be one of the determinants for the synergism between biguanide and erlotinib combination, and such synergism can be applied to cancer types other than breast cancer.

### Combination treatment of metformin and erlotinib induces tumor regression in a mouse orthotopic transplant model

**Figure 5:**
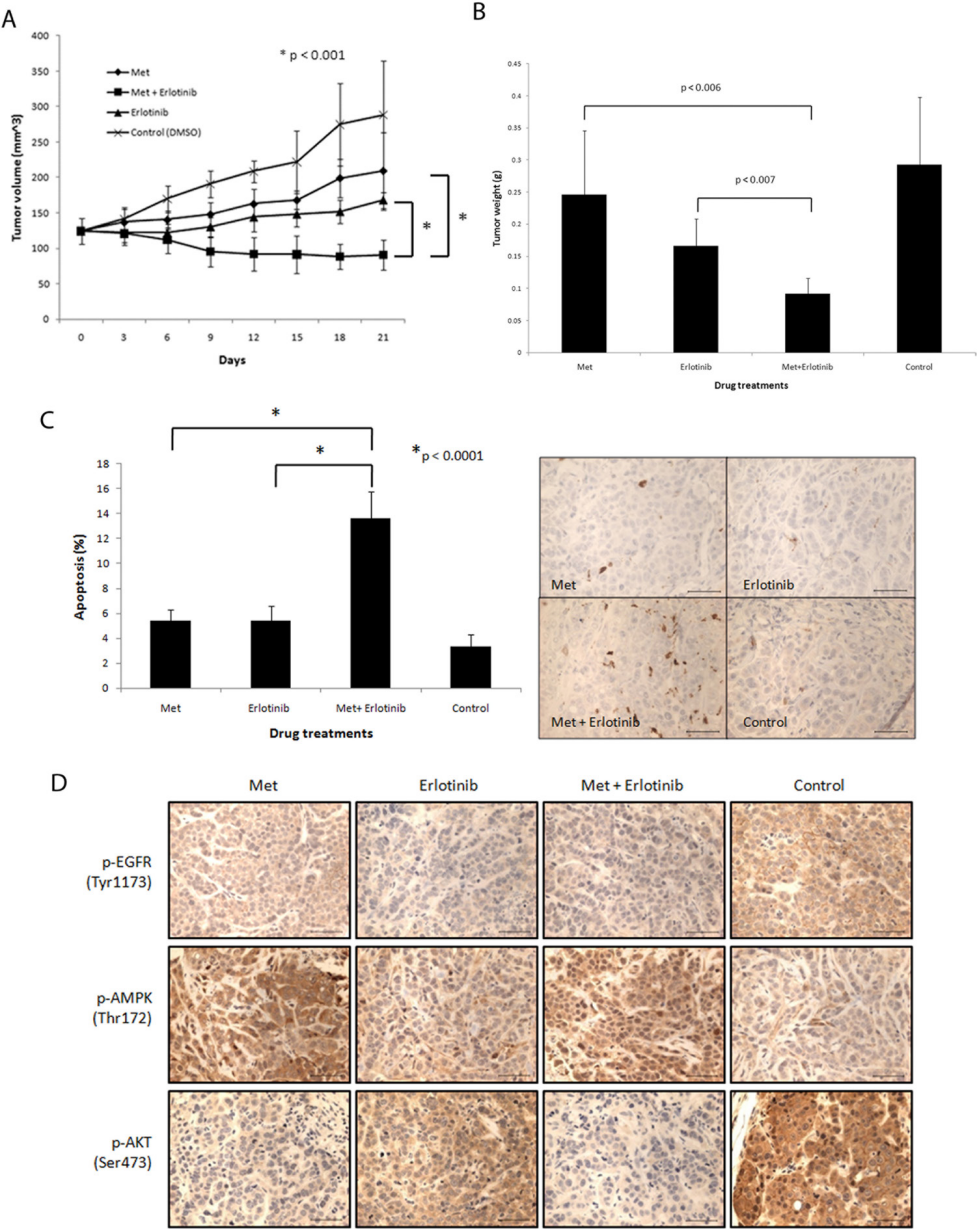
Combined treatment of metformin and erlotinib induces tumor regression, inhibits drug targets *in vivo* and induces apoptosis in MDA-MB-468 xenograft model **(A)** Tumors formed by MDA-MB-468 cells in mice were treated with metformin (250 mg/kg/day), erlotinib (50 mg/kg/day) and their combination via oral gavage 6 days a week for 21 days. Tumor growths were monitored over 21 days by caliper measurements every 3 days. A minimum of 6 tumors were used per arm. **(B)** Tumors were excised 24 hours after last drug treatments and final tumor weights were obtained. **(C)** Apoptosis in tumors excised 24 hours after last drug treatments were assessed by immunohistochemistry (IHC) using cleaved caspase 3 antibody and results were quantitated (6 or 7 samples per treatment group). 3 views were counted per sample. Length of scale bar equals 50 μm. **(D)** Signaling status of EGFR, AKT and AMPK in tumors excised 24 hours after last drug treatments was assessed by IHC. Length of scale bar equals 50 μm.

Next, we assessed the efficacy of the combination treatment *in vivo* using an orthotopic transplant MDA-MB-468 cell xenograft model. The combination treatment resulted in tumor regression (28% decrease by volume) while the individual treatments each only marginally slowed tumor growth (68% increase by volume in the cohort treated by metformin and 35% increase by volume in the cohort treated by erlotinib) over a course of 21 days (Fig. 5A). Tumor weights at the end of the experiment confirmed a significant reduction of tumor burden (69% reduction compared with control) with the drug combination as compared with single agent treatments (Fig. 5B). We did not observe any toxicity in any of the cohorts. Measured plasma trough levels of both drugs taken at the time the tumors were harvested (24 hours after their last treatment) were low [metformin (0.17 μM) and erlotinib (0.12 μM)]. We asked if altered circulating insulin played a role in the observed tumor regression but found that trough plasma insulin levels were not significantly different between the cohorts (Suppl. Fig. 8). Combined treatment using a 5-fold lower dose of metformin (50 mg/kg/day) also inhibited xenografted PTEN null HCC-70 BBC cells (Suppl. Fig. 9). To thoroughly understand the drug concentration in plasma, we treated mice with metformin 50 mg/kg and 50 mg/kg erlotinib and conducted a pharmacokinetics study (Suppl. Fig. 10). For erlotinib the Cmax was 7283 ± 1344 ng/mL (18.5 uM) and the trough level was < 0.25 ng/ml with a terminal half-life of 1.5 hours. For metformin, the Cmax was 1828 ± 259 ng/mL (14.2 uM), the trough level was 2.9 ± 0.4 ng/ml and the terminal half-life was 3 hours. There was no accumulation of either drug consistent with pulse daily dosing (see supplemental PK data for full modeling details).

### Metformin and erlotinib combined to inhibit cell signaling and induce apoptosis in vivo

Tumors were harvested 24 hours after their last treatment and assessed by immunohistochemcial staining. The apoptosis marker, cleaved caspase 3, revealed significantly more apoptosis (13.6%) in the combined treated tumors (Fig. 5C). On the other hand, Ki-67 and phospho-Histone H3 staining showed that proliferation was not preferentially affected (data not shown). As expected, control tumors showed very high activation of EGFR as shown by intense membranous staining of p-EGFR at the Tyr1143 residue. Erlotinib alone, and metformin alone (to a lesser extent), reduced EGFR phosphorylation, while the combined treatment completely inhibited EGFR phosphorylation (Fig. 5D; Suppl. Fig. 11). Control tumors showed almost no AMPK phosphorylation while metformin, as shown in both single and combined treatments, significantly activated AMPK. We found highly phosphorylated AKT in the control tumors as shown by intense membranous staining (Fig. 5D). Erlotinib alone partially reduced AKT phosphorylation and surprisingly, metformin alone also significantly inhibited AKT phosphorylation in these tumors. Importantly, we observed almost complete inhibition of AKT phosphorylation in tumors treated with the combined therapy. These signaling changes were all consistent with what was observed *in vitro*. These results show that the combination of metformin and erlotinib synergize to trigger significant apoptosis that neither drug alone can achieve, while collaborating to inhibit both upstream and downstream PI3K pathway signaling.

## DISCUSSION

To date, no effective targeted therapies are available for BBCs. The catalog of documented genetic alterations found in BBC tumors, with many occurring concurrently, suggests that single agent targeted therapies are unlikely to be effective in most patients. We show, for the first time, that metformin can synergize with a targeted drug, erlotinib, to induce tumor regression in an orthotopic transplanted BBC model while demonstrating that loss of PTEN is one potential marker of sensitivity.

We demonstrated that metformin and erlotinib can have synergistic or additive effects in a significant subgroup of BBC cell lines. It was encouraging to find that in most of the “synergistic” cases, the synergy was caused, at least in significant part, by induction of apoptosis. Our screen across a panel of 17 breast cancer lines with known PTEN mutation status and differential effects seen in the isogenic cell lines with and without PTEN expression are evidence that PTEN is a candidate biomarker of sensitivity towards the drug combination. PTEN expression was present among all the cell lines in the antagonistic group with the exception of HCC-38. Possible explanations for the lack of synergy in PTEN-null HCC-38 cells include 1) the fact that it harbors a fusion gene comprised partly of the *SLC22A1* gene (which codes for OCT1) fused with *CUTA* (from the Sanger mutation database), 2) it has the lowest level of phosphorylated EGFR among the 17 cell lines (data not shown) and, 3) existence of other unknown genetic alterations in the relevant signaling pathways. Further investigation of the mechanism of resistance towards the combination treatment in HCC-38 cells is warranted.

Since loss of PTEN increases the synergy of the drug combination and gain of PTEN decreases the synergy, the synergy is likely in part due to activation of the PI3K pathway and thus dependent on a subset of its signal outputs. Inhibition of one PI3K output, mTOR, has previously shown to synergize with EGFR inhibition [42] so one obvious explanation for the observed synergy is metformin's ability to directly and indirectly inhibit mTOR [30, 31]. Although we also confirmed the ability of metformin to inhibit S6 and 4EBP1 phosphorylation independently of AMPK activation, the observation that erlotinib in combination with metformin outperformed erlotinib in combination with either rapamycin or AZD8055 (TORC kinase inhibitor) suggests that additional factors are contributing to our observed synergy. One observed difference between combining erlotinib with metformin as opposed to specific mTOR inhibition was a significant additional decrease in EGFR phosphorylation. This potentiation of the inhibitory effect of erlotinib on EGFR by metformin was also not observed when erlotinib was combined AICAR so it is unlikely due to activation of AMPK. These observations are novel and require further study. We also showed that metformin treatment combined with either an AKT or MEK inhibitor in place of erlotinib generated an inferior inhibitory effect. These results may be explained by the fact that there was no observed stimulatory feedback when erlotinib was combined with metformin. In fact, the combination of metformin and erlotinib resulted in potentiation of decreased AKT and EGFR phosphorylation instead of previously described feedback resulting in increased phosphorylation of AKT with rapamycin treatment or increase EGFR phosphorylation with AKT inhibitor treatment [43, 44]. No other signaling pathway alterations were detected in RTK antibody array.

Data from TCGA confirms that activation of the PI3K-AKT pathway is high in the majority of BBCs as compared with other subtypes of breast cancer [1]. Using the cBIO Cancer Genomic Portal, we confirmed the well-established enrichment of relatively increased EGFR mRNA in BBC [RNAseq z-score > 1 in 14/81 (28%) of BBC versus 6/324 (2%) of luminal cases. p < 0.0001 by Fisher Exact Probability Test] [37]. More importantly, 21 out of 23 of the BBC with “high” EGFR mRNA levels have a concurrent alteration in the PI3K pathway (PTEN, INPP4B, AKT1/2/3, or PIK3CA; data not shown). Instead of *PTEN* mutation, BT-20 (a basal line) and MCF7 (a luminal line) cells in the “synergistic group” harbor *PIK3CA* mutations and increased phosphorylation of EGFR. Thus it is reasonable to suggest that erlotinib and metformin could have therapeutic potential in any breast cancer driven by activated EGFR and PI3K pathways. Importantly, we observed in our short panel of breast cancer cell lines that although expression of EGFR was much lower in the luminal cell lines, the phosphorylation status was very comparable to that of the basal-like lines. This observation is consistent with the TCGA proteomic breast cancer data in which, despite the difference in total EGFR mRNA levels between basal and luminal cases, levels of phosphorylated EGFR between basal and luminal subtypes were similar (26% of basal and 24% of luminal case with p-EGFR protein z-scores > 1) [37]. One could further speculate that the synergy between EGFR inhibition and metformin may occur in other tumor types enriched for concurrent EGFR and PI3K pathway activation, including squamous cell cancers, colorectal cancer, glioblastoma with *EGFR* vIII amplification, and lung cancer with *EGFR* exon 18 through 21 mutations. In fact, we detected synergistic cell death from three other cancer lines H4, CAOV-3 and NCI-H1975, which harbor either PTEN or PI3KCA mutation and EGFR amplification or mutation. There is a recent study showing that metformin synergizes with another EGFR inhibitor gefitinib in lung cancer cell lines [45].

We also investigated if LKB1, upstream of AMPK, played any role in the observed synergy. Reducing the LKB1 protein level via shRNA in MDA-MB-468 cells showed no effect on synergy towards the drug combination (data not shown) suggesting that either metformin requires only a small amount of basal LKB1 to activate AMPK or metformin's antineoplastic effects are largely independent of LKB1. Our cell signaling data suggest that the synergy is independent of LKB1 and is further supported by our observation that the drug combination has an additive effect on SUM-149 cells, which lacks LKB1 expression. Our data are consistent with the previous observation that metformin and phenformin were able to delay outgrowth of PTEN deficient and LKB1 hypomorphoic xenografts in the absence of significant changes in insulin levels [32].

It has been shown that metformin can inhibit tumor initiating cells and prevent relapses *in vivo* when combined with chemotherapy [21]. In our study, we found that metformin or erlotinib alone can inhibit mammosphere formation in multiple BBC cell lines. This confirms previous findings that EGFR inhibition leads to reduced mammosphere formation in breast cancer cells when co-cultured with mesenchymal stem cells [46]. We showed that combining the two drugs was significantly more potent compared with either alone and completely prevented mammosphere formation in multiple BBC cell lines. The reason for this result warrants further investigation and supports the hypothesis that the drug combination may help prevent relapses in BBC patients.

It is controversial whether the effects of metformin at high concentrations have translational value. Metformin was used at doses of 0.5 to 4 mM in many of our *in vitro* studies. Although much higher than the plasma level in patients (about 10 μM) [47], these doses are lower than most of the published *in vitro* data, with the notable exception of metformin's ability to inhibit tumor initiating cells [21]. AMPK activation was demonstrated in muscle biopsies from patients given typical diabetic doses of metformin which agrees with our data showing that much lower levels (micromolar) of metformin are needed *in vivo* to obtain the same cell signaling changes which require millimolar concentrations *in vitro* [48]. Increased p-AMPK (Thr 172) immunohistochemical staining was also seen in the HCC-70 xenografted tumors treated with 50 mg/kg/day of metformin (data not shown). Therefore, the higher concentrations of metformin needed for *in vitro* experiments are likely, at least in part, due to artifacts of non-physiologic tissue culture conditions [27]. Recently there have been two reports showing that intermediate concentrations of metformin (300–500 μM) can reprogram cancer cell metabolism [49, 50]. Furthermore, we had the observation that glucose levels can affect the inhibitory effect of metformin on breast cancer cells *in vitro* as others have observed in other settings [Fig. 3G; [27, 38, 39]]. Under physiological level of glucose (5 mM), we still observed similar level of synergism. Although cells in low glucose medium are more sensitive to treatment, the synergism diminishes compared to normal glucose level.

There is also evidence that metformin can accumulate within tissues leading to higher concentrations than plasma levels [51]. Our immunohistochemistry results demonstrate that the drug-induced signaling alterations of EGFR, AKT, and AMPK were maintained 24 hours after administering the last dose of metformin and erlotinib, corresponding to the concurrently measured low micromolar plasma levels of each drug, which is consistent with their reported half-lives in mice of about 3 hours. We did not observe differences in plasma insulin levels between the mouse cohorts, suggesting that the observed signaling effects were due to a direct interaction of the drugs with tumor cells. However, the potential effects of altered growth factor levels by metformin cannot be completely discounted by our data, especially given the rapid kinetics of growth factor levels (e.g. the half-life of insulin in mice is 10 min) [52]. Despite the limitations of our studies, our aggregate observations, and the ability of the drug combination to induce apoptosis with resulting tumor regression *in vivo*, demonstrates translational potential.

Our study provides evidence that combination therapy with metformin and erlotinib could have therapeutic efficacy in cancers driven by EGFR and PI3K signaling, including a subset of BBC patients, and provides a rationale for clinical study. Both metformin and erlotinib are well-tolerated orally administered FDA-approved drugs that can be easily translated into clinical trials and could serve as a platform for the addition of other targeted drugs and chemotherapy.

## STATEMENT OF SIGNIFICANCE

BBCs make up the large majority of the triple negative clinical subtype of breast cancers (TNBCs) and are aggressive tumors deficient of effective targeted therapies. Our *in vitro* and *in vivo* data show that combining metformin with erlotinib may be considered in strategies to treat TNBCs, with loss of PTEN expression as one candidate biomarker of sensitivity.

## METHODS

### Human breast cell lines

MDA-MB-468, MDA-MB-157, MCF7, MDA-MB-435S, BT-20, MDA-MB-436, MX-1, L56Br-C1, MDA-MB-231, and CAOV-3cells were obtained from American Type Culture Collection (ATCC) and cultured in DMEM media and 10% fetal bovine serum (FBS). HCC-1143, HCC-1806, HCC-1937, HCC-70, HCC-38, HCC-1187, BT-549 cells were obtained from ATCC and cultured in RPMI-1640 media and 10% FBS. Glioma cell line H4 was kindly gave to us by Dr. Richard Baer (Columbia University) and cultured in DMEM media and 10% FBS. Lung cancer cell line NCI-H1975 was kindly gave to us by Dr. Balazs Halmos (Columbia University) and cultured in RPMI-1640 media and 10% FBS. SUM-149 cells were cultured in HAM's F12 media and 10% FBS supplemented with insulin (10 μg/ml) and hydrocortisone (500 ng/ml). HMEC-hTERT cells were kindly given to us by Dr. Robert Weinberg and cultured in DMEM/F12 media supplemented with EGF (10 ng/ml), hydrocortisone (250 ng/ml) and insulin (10 μg/ml). MCF10A parental cells and MCF10A PTEN-null cells were kindly given to us by Dr. Kurtis Bachman [53]. Derivatives were made by infecting MCF10A PTEN-null cells with retroviral vector pBABE-DDp53-hyg (Addgene plasmid 9058) and pBABE-EGFR-puro (Addgene plasmid 11011) as described in [41]. All MCF10A lines were cultured in DMEM/F12 media and 5% horse serum supplemented with EGF (20 ng/ml), hydrocortisone (500 ng/ml), insulin (10 μg/ml) and cholera toxin (100 ng/ml). All cells were cultured with 1% penicillin/streptomycin at 37^o^C with 5% CO_2._

### Reagents

Metformin and phenformin were obtained from Sigma-Aldrich. Erlotinib and rapamycin were obtained from LC Laboratories. AICAR was obtained from Cell Signaling. BIBW2992 and AZD8055 were obtained from Selleck Chemicals. MK2206 and GSK1120212 were obtained from the Stand Up To Cancer drug inventory.

### Antibodies

Antibodies obtained from Cell Signaling Technologies: Anti- phospho-AMPK(T172), pan AMPKα, phospho-ACC(S79), phospho-EGFR(Tyr1173), phospho-S6(S235/236), phospho- S6(S240/244), phospho-4EBP1(T70), 4EBP1(53H11), phospho-Erk1/2 (Thr202/Tyr204), Erk1/2, phospho-AKT(S473), AKT, cleaved caspase 3, LKB1(27D10), PTEN(138G6), BIM (C34C5) and phospho-Histone H3. Antibodies obtained from Santa Cruz: EGFR, S6. Anti-phospho-EGFR(Tyr1173) and anti-AMPK-a1 antibodies were obtained from Millipore. Anti- SLC22A1 (OCT1) and Ki-67 antibodies were obtained from Abcam. Anti-SLC22A2 (OCT2) antibody was obtained from Sigma-Aldrich. Anti-p53 antibody was obtained from Calbiochem.

### Analysis of cytotoxic interactions between metformin and erlotinib

Each cell line seeded at 10,000 cells/well in 48-well plates was treated with the following drug treatments: metformin (0.5, 1, 2, 4 mM), erlotinib (2, 4 μM) and combination of metformin and erlotinib at all these doses. 6 replicates were used for each treatment. Cell viability was assessed by crystal violet assay after 6 days of drug treatments. The combination index (CI) reflecting additive (0.8–1.2), synergistic (< 0.8) and antagonistic (>1.2), was obtained by using the software Compusyn (version 3.01). We have modified the stringency of the analysis by defining “additive” as cell lines having CI between 0.8 and 1.2 instead of exactly at 1. This experiment was also carried out in PTEN-null MDA-MB-468 cells and HCC-1937 cells retrovirally transfected with the plasmids 1066 pBabe puroL PTEN (plasmid #10785 from Addgene) and pBabe-puro (plasmid #1764 from Addgene).

### Western blot analysis

Cells were lysed with 2X Laemmli buffer. 25 μg of total proteins were separated by electrophoresis and transferred to polyvinylidene fluoride membranes. Membranes were incubated with primary antibodies followed by secondary antibodies and then developed with an enhanced chemiluminescence detection kit according to manufacturer's instructions.

### Proliferation assay

6-well proliferation assay: 400,000 cells/well were seeded, treated every 2 days and grown for 6 days. Cell number was obtained every 2 days. 48-well proliferation assay: 10,000 cells/well were seeded, treated every 3 days and grown for 6 days. Cells were stained with 0.05% crystal violet solution at the end of the experiment and absorbance was measured at 590 nm.

### Cytotoxic clonogenic assay

Cells were seeded in 6-well plate at a sparse density of 800 cells/well and treated with various drugs (metformin = 0.5 mM, erlotinib = 4 μM, rapamycin = 10 nM) and their combinations for 3 days. Colonies were counted after culturing for 2 weeks.

### RTK signaling antibody array

MDA-MB-468 cells were seeded in 6-well plate and treated with vehicle control (Ctrl), metformin (1 mM), erlotininb (4 μM) and their combination for 24 hours. The samples were processed according manufacture's protocol (Cell Signaling).

### Mammosphere formation assay

Cells were seeded at 20,000 cells/well in ultralow attachment 6-well plate in DMEM/F12 media supplemented with B27, EGF (20 ng/ml), insulin (10 μg/ml) and hydrocortisone (70 ng/ml) and allowed to grow for 6 days. The majority of cells could not survive under non-attachment conditions and only cells capable of forming mammospheres proliferated. The number of mammospheres was counted 6 days after the start of the drug treatments.

### Flow Cytometry

Cells were seeded at 60,000 cells/well and treated with metformin (0.5, 1 or 2 mM) or erlotinib (4 μM) and their combination. Cells were then trypsinized and stained with both FITC Annexin V antibody and propidium iodide (PI) to assay for cell death after 3 days using the FITC Annexin V Apoptosis Detection Kit I (Becton Dickenson). Procedures were carried out according to manufacturer's instructions. Fluorescence-activated cell sorting (FACS) analysis was performed on a BD FACSCalibur flow cytometer (Becton Dickenson) and the results were analyzed using the software FlowJo (Tree Star Inc).

### *In vivo* drug treatment study

All animal experiments were conducted according to protocols approved by the Institutional Animal Care and Use Committee (IACUC) at Columbia University Medical Center. 1 million MDA-MB-468 cells mixed with Matrigel were injected into the mammary fat pad of each mouse and allowed to grow into tumors with size of approximately 125 mm^3^. The mice were randomized into 4 groups (6 to 7 animals per group) and treated with metformin alone (250 mg/kg/day), erlotinib alone (50 mg/kg/day), metformin and erlotinib, or DMSO through daily oral gavage (6 days per week). Tumor size was measured every 3 days up to 21 days. 24 hours after the final drug treatments, tumors were excised and weighted by a digital scale. Trough plasma levels of metformin and erlotinib were assessed by ultra performance liquid chromatography and tandem mass spectrometry using a Zorbax Eclipse XDB C18 column, an Agilent 1290 UPLC and an Agilent 6410 triple quadrupole mass spectrometer. Cmax levels of metformin and erlotinib were simulated based on individualized compartmental pharmacokinetic parameters for each drug derived from earlier reports [51, 54] using a model developed previously [55] and the MW-Pharm pharmacokinetic software (Mediware). Plasma insulin level was measured by ALPCO Insulin Assay according to manufacturer's instructions (ALPCO Diagnostics). Excised tumors were fixed in 10% formalin and stained with various antibodies for immunohistochemical analysis.

### Pharmacokinetics study

A limited sampling method and modeling was used. Groups of 3 mice each were treated with metformin (50 mg/kg/day) and erlotinib (50 mg/kg/day) by oral gavage for 6 out of 7 days for 3 weeks total. Mice were sacrificed for plasma collection at the following time-points: Day 1 [1 hr, 2 hr, 4 hr, 8 hr and 24 hr (day 2 trough)], immediately prior to the day 6 dose [day 6 trough], one hour after the day 6 dose [day 6 peak], and 4 hours after the last treatment. See supplemental PK methods and results for full details.

### Statistics

Student's two-tailed t-test was used to analyze statistical differences between experimental groups.

## SSUPPLEMENTARY FIGURES AND MODELING


