# Supplementary material for: Metformin and erlotinib synergize to inhibit basal breast cancer
Source: Oncotarget. 2014 Nov 4;5(21):10503–17. doi: 10.18632/oncotarget.2391 (PMC4279389; doi:10.18632/oncotarget.2391)
Supplement: Supplementary file 1 [file oncotarget-05-10503-s001.pdf]

SUPPLEMENTARY FIGURES AND MODELING

Metformin and Erlotinib PK modeling

Metformin pharmacokinetics looks straightforward and is characterized by 2-compartment pharmacokinetics. After a single dose of Metformin (50 mg/kg) + Erlotinib (50 mg/kg) tmax = 1h and Cmax = 1828 ± 259 ng/mL [14.153 micromolar], which is similar to C1h after 1 week of treatment (1464 ± 515 ng/mL). C4h after a single administration of the combination is 415 ± 285 ng/mL. After 3 weeks of treatment, C4h is approximately the same whether treated with the combination (427 ± 98 ng/mL) or with metformin only (290 ± 75 ng/mL). C24h after a single administration of Metformin (50 mg/kg) + Erlotinib (50 mg/kg) is 2.9 ± 0.4 ng/mL. After 1 week of treatment C24h is unchanged (3.7 ± 1.0 ng/ml). This data suggest there is little to no accumulation of metformin in plasma. The data is adequately described by a 2-compartment model with first order absorption: F=1, k_a=2.71h^{-1}, k_{elm}=1.11h^{-1}, V_1=9.17L, k_{12}=1.20h^{-1}, k_{21}=0.52h^{-1}, t½(1)=0.3h, t½(2)=3.0h. Figure 1 shows the measured and simulated plasma concentrations of metformin during the combination treatment regimen, which consisted of 50mg/kg Metformin and 50mg/kg Erlotinib for 3x6 consecutive days interrupted by one day without treatment.

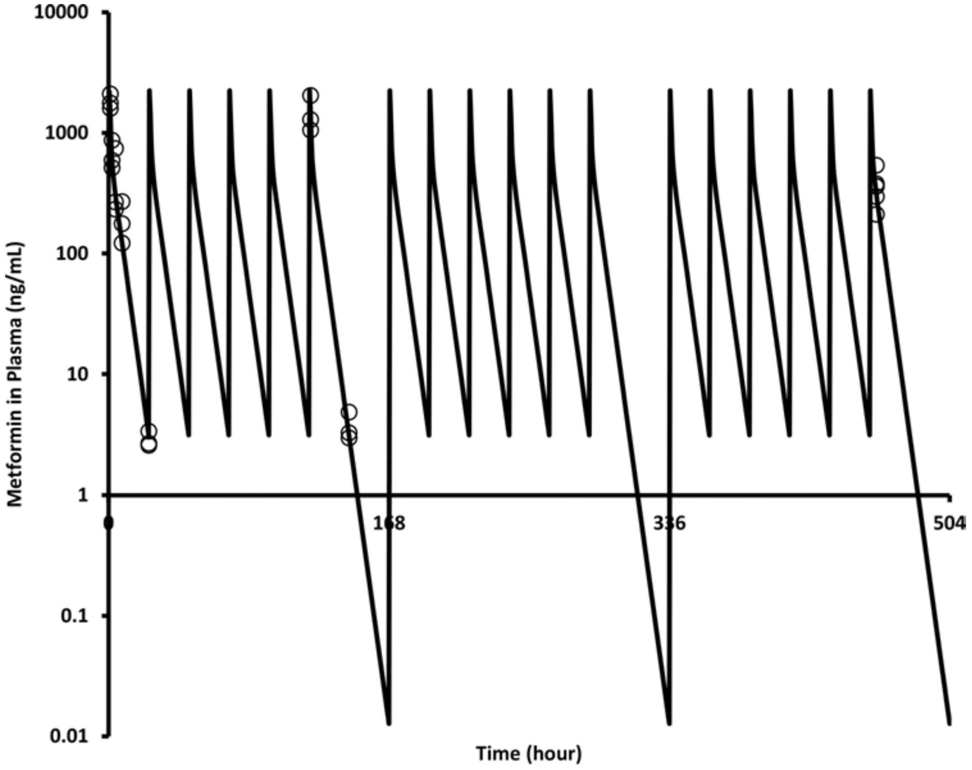

Erlotinib pharmacokinetics also looks straightforward and is characterized by 2-compartment pharmacokinetics. After a single dose of Metformin (50 mg/kg) + Erlotinib (50 mg/kg) tmax = 1h and Cmax = 7283 ± 1344 ng/mL [18.5113 micromolar], which is similar to C1h after 1 week of treatment (7271 ± 2733 ng/mL). C4h after a single administration of the combination is 5188 ± 1117 ng/mL. After 3 weeks of treatment, C4h is approximately the same whether treated with the combination (4825 ± 797 ng/mL) or with erlotinib only (5100 ± 2304 ng/mL). C24h after a single administration of Metformin (50 mg/kg) + Erlotinib (50 mg/kg) is <0.25 ng/mL. After 1 week of treatment C24h seems unchanged (<0.5 ng/ml), although all erlotinib data from both time points were below the limit of quantification of the assay. This data suggest there is little to no accumulation of erlotinib in plasma. The data is adequately described by a previously described multi-compartment model for erloninib and its metabolite OSI-420 (Elmeliegy et al. Clin Cancer Res 2011;17:89–99): The PK parameters of the model calculated from the current data are: F=1, k_a=0.52h^{-1}, k_{24}=97.5h^{-1}, k_{42}=0.63h^{-1}, V_2=0.0028L/kg, k_{23}=523.7h^{-1}, k_{35}=0.079h^{-1}, k_{53}=0.359h^{-1}, k_{30}=53.5h^{-1}, t½ terminal=1.5h. Figure 2 shows the measured and simulated plasma concentrations of erlotinib during the combination treatment regimen, which consisted of 50mg/kg Metformin and 50mg/kg Erlotinib for 3x6 consecutive days interrupted by one day without treatment.

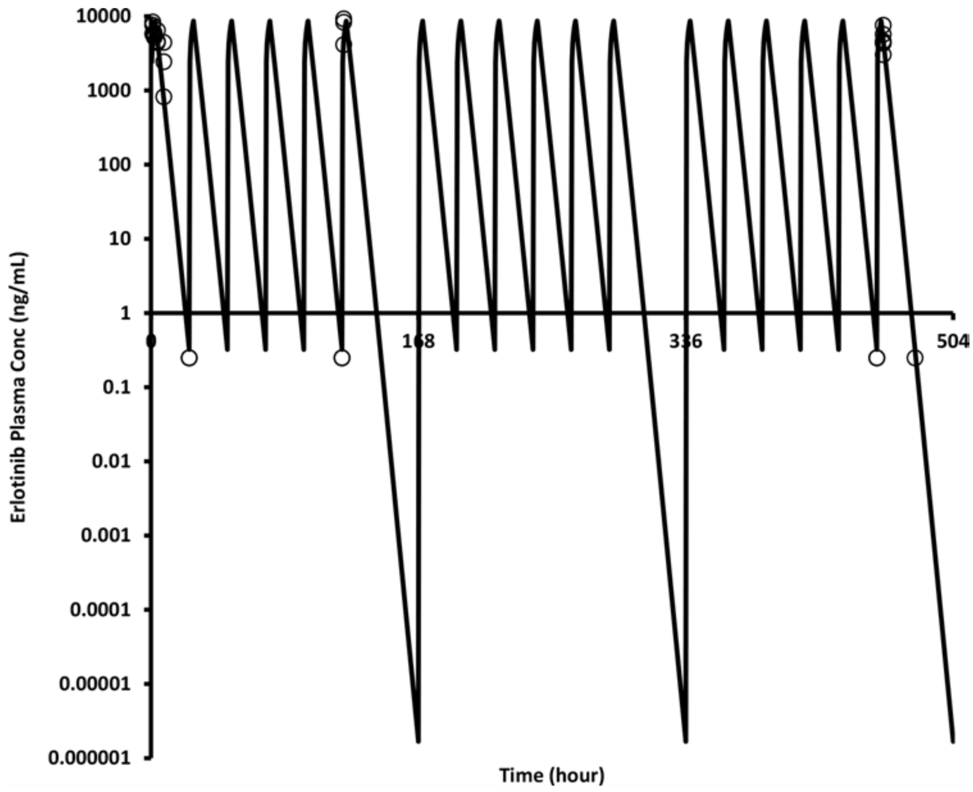

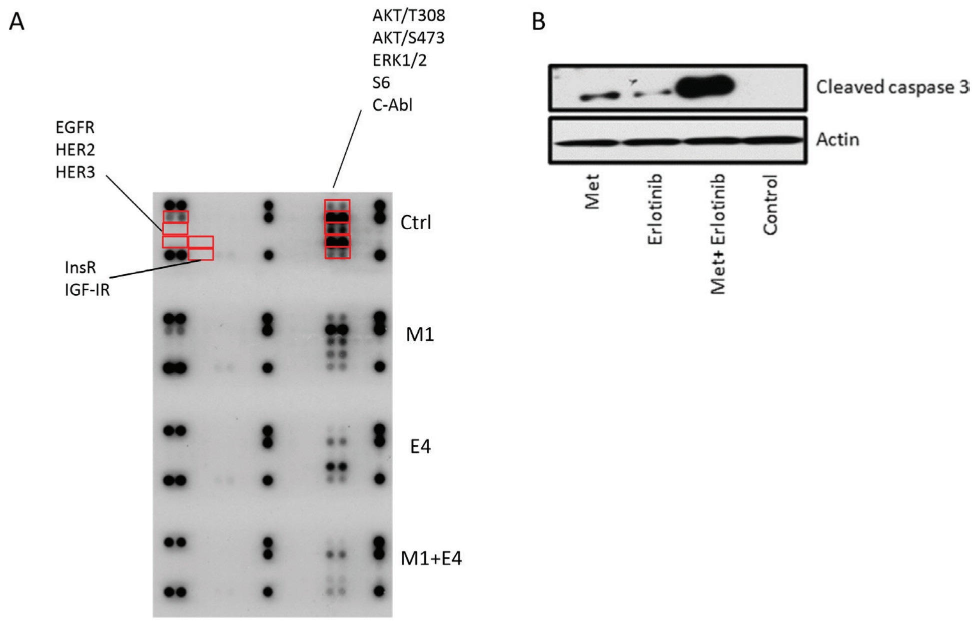

Supplementary Figure S1: Other signaling pathways alteration is not detected and combined treatment with metformin and erlotinib triggers cleavage of caspase 3 in MDA-MB-468 cells. (**A**) RTK signaling antibody array of MDA-MB-468 cells treated with vehicle control (Ctrl), 1 mM metformin (M1), 4 uM erlotinib (E4) and their combination (M1+E4) for 24 hours. The samples were processed according manufacture's protocol (Cell Signaling). (**B)** Immunoblot analysis of cleaved caspase 3 of cells treated with metformin (2 mM), erlotinib (4 µM) and their combination for 48 hours.

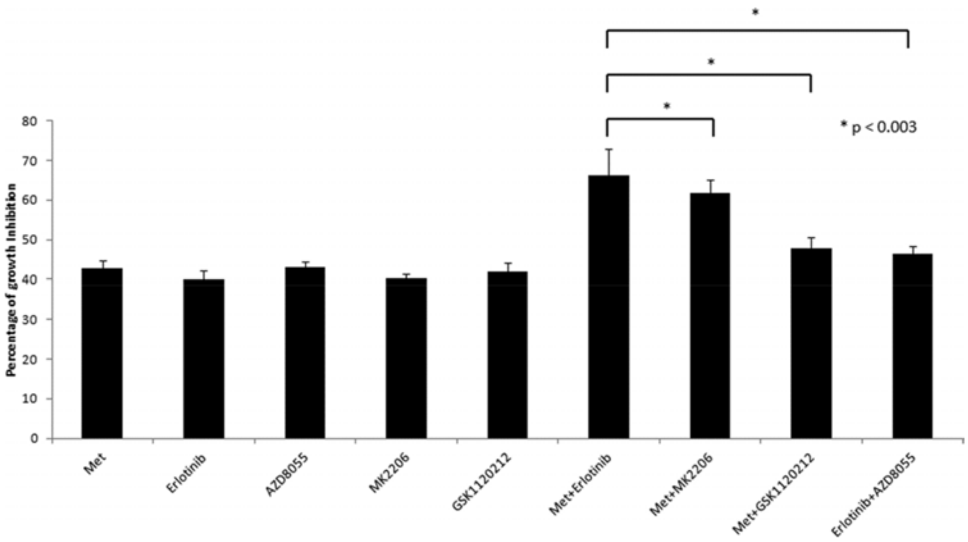

Supplementary Figure S2: Combined treatment with metformin and Erlotinib exhibits greater synergy in MDA-MB-468 cells than when they were combined with AKT, MEK and mTOR inhibitors. MDA-MB-468 cells were seeded at 10,000 cells per well in 48-well plate and treated with various drugs with their respective IC40 concentrations [metformin (2 mM), erlotinib (2 µM), AZD8055 (32 nM), MK2206 (1.7 µM), GSK1120212 (40 nM)] and a few of their combinations (metformin with erlotinib; metformin with MK2206; metformin with GSK1120212; erlotinib with AZD8055) over 6 days. 6 replicates were used per treatment. Cell viability was analyzed by measuring intensity of crystal violet staining at wavelength of 570 nm using a microplate reader.

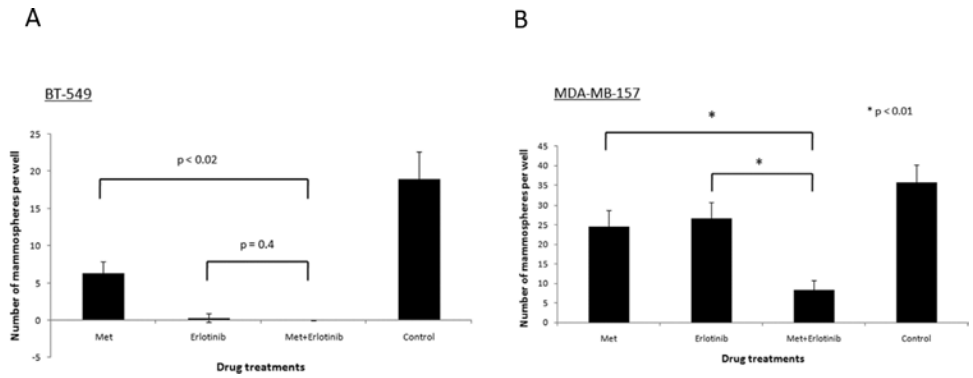

Supplementary Figure S3: Combined treatment with metformin and erlotinib inhibits mammospheres formation in BT-549 and MDA-MB-157 cells. Metformin and erlotinib inhibits mammospheres formation in **(A)** BT-549 **(B)** MDA-MB-157. Cells were seeded at 20,000 cells/well in ultralow attachment 6-well plate with special media without serum and treated with metformin (2 mM), erlotinib (4 µM) and their combination. The number of mammospheres was counted 6 days after the start of drug treatments.

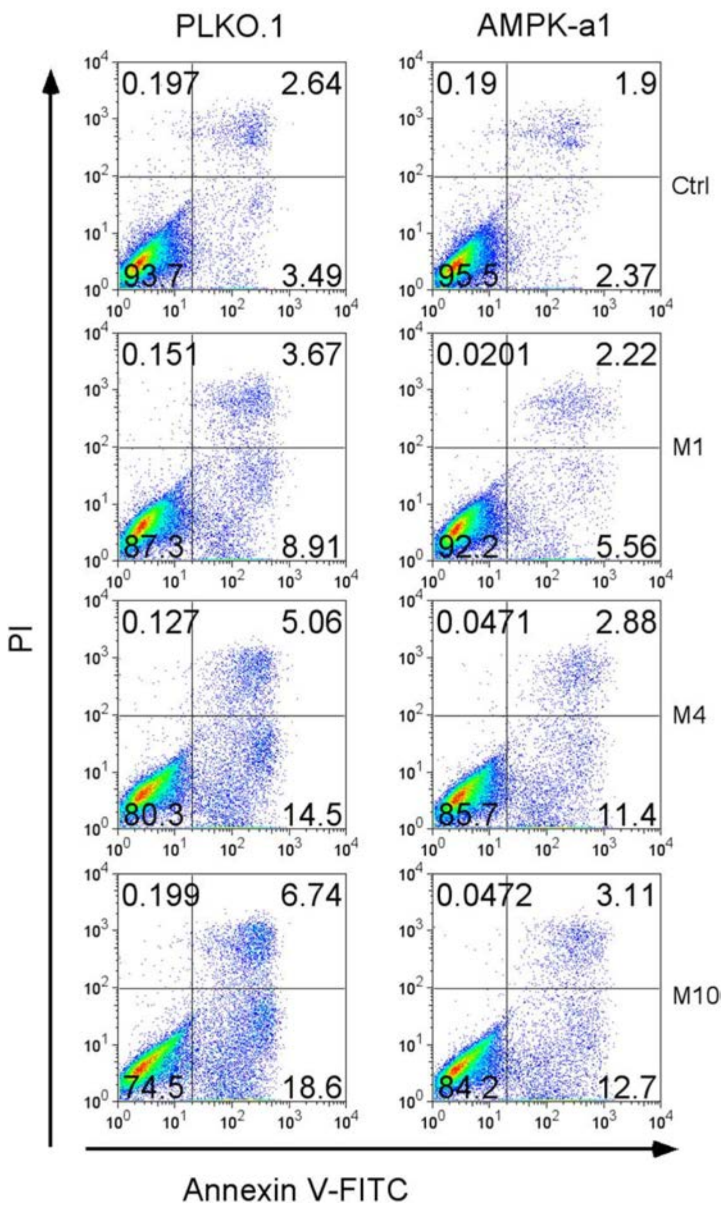

Supplementary Figure S4: MDA-MB-468 cells with AMPK-a1 knockdown are resistant to metformin treatment. MDA-MB-468 cells were transduced with scramble PLKO.1 and AMPK-a1 shRNA lentiviruses and subjected to puromycin. Cells were further treated vehicle control (Ctrl), 1 mM metformin (M1), 4 mM metformin (M4), 10 mM metformin (M10) for 72 hr. Apoptotic cells were detected with Annexin-V FITC and PI staining.

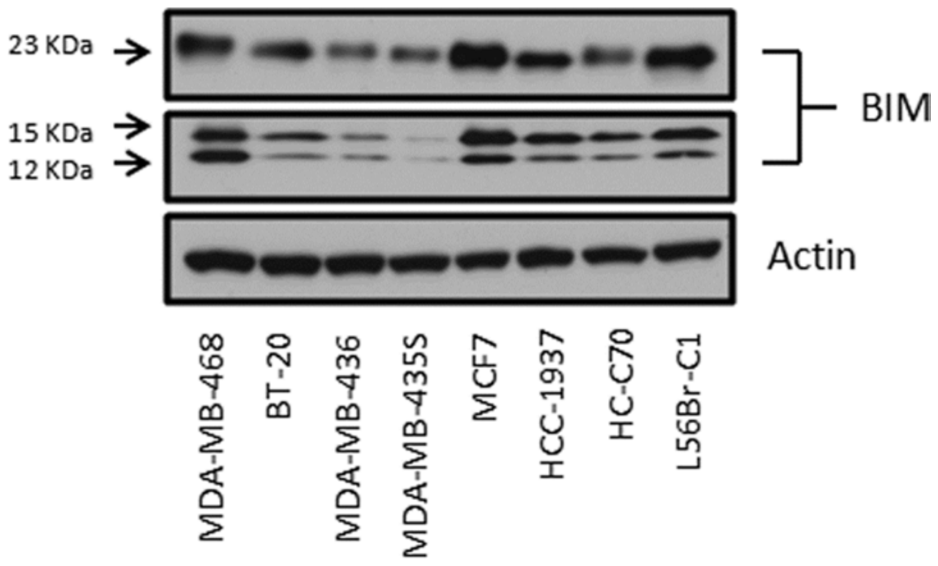

Supplementary Figure S5: MDA-MB-436 cells and MDA-MB-435S cells show lowest endogenous expression of BIM proteins among 8 cell lines that show significant cell death under combined metformin and erlotinib treatment. Immunoblot analysis of endogenous levels of 3 BIM species (23 kDA, 15 kDa, 12 kDa) expressed by 8 cell lines when they were at 80% confluency culturing conditions.

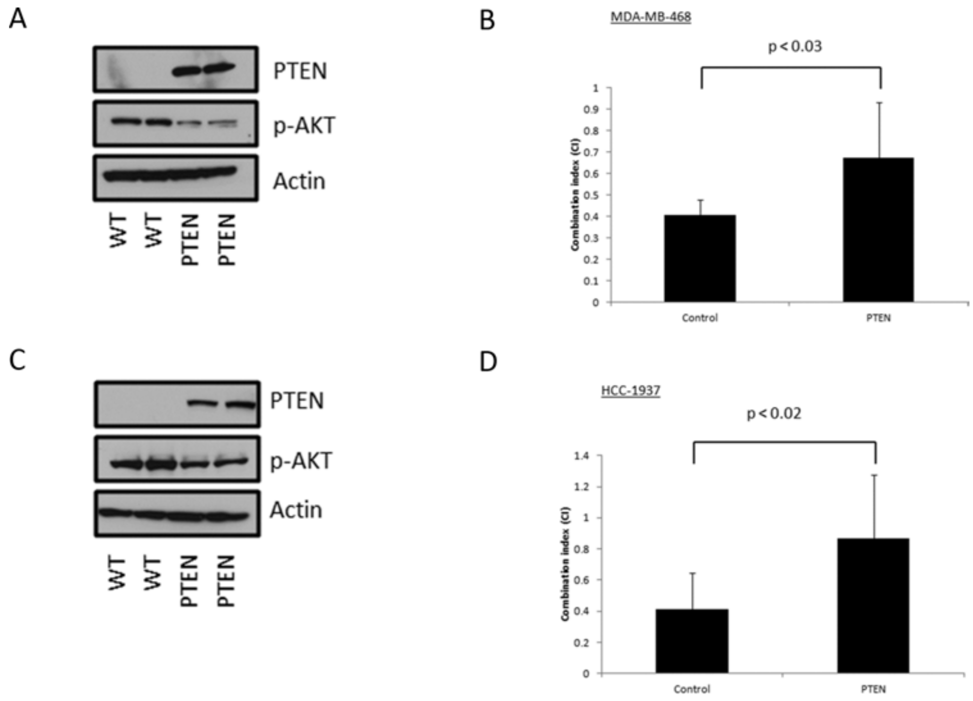

Supplementary Figure S6: Expression of PTEN in PTEN-null breast cancer cell lines decreases synergy of metformin and erlotinib as measured by combination index (CI). (A) Immunoblot analysis of PTEN expression and phosphorylation of AKT in wild type (WT) and PTEN-expressing MDA-MB-468 cells. **(B)** MDA-MB-468 cells were seeded at 10,000 cells per well in 48-well plate and subjected to drug treatment of metformin (0.5, 1, 2, 4 mM) and Erlotinib (2 and 4 µM) and their combinations over 6 days. 6 replicates were used per condition. Cell viability was analyzed using crystal violet staining. Combination indices (CI) were determined by using the Compusyn software. **(C)** Immunoblot analysis of PTEN expression and phosphorylation of AKT in wild type (WT) and PTEN-expressing HCC-1937 cells. **(D)** HCC-1937 cells were seeded at 10,000 cells per well in 48-well plate and subjected to drug treatment of metformin (0.5, 1, 2, 4 mM) and Erlotinib (2 and 4 µM) and their combinations over 6 days. 6 replicates were used per condition. Cell viability was analyzed using crystal violet staining. Combination indices (CI) were determined by using the Compusyn software.

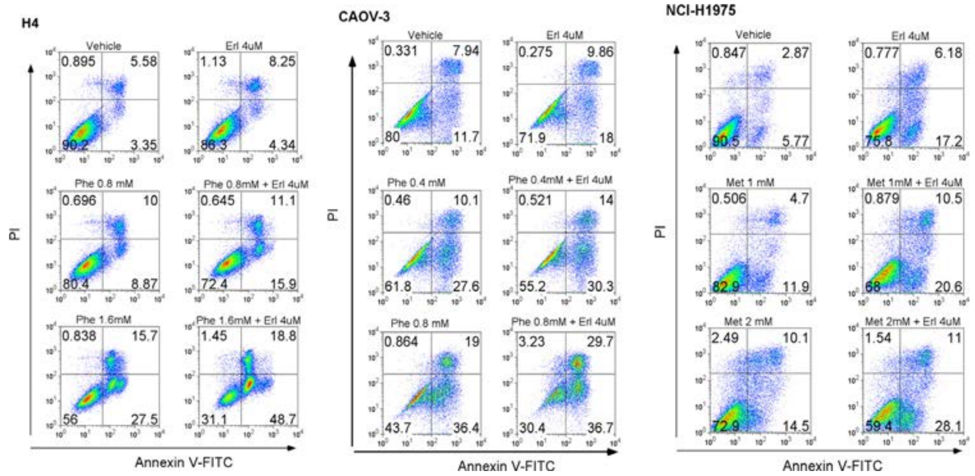

Supplementary Figure S7: Synergistic effect of metformin/phenformin with erlotinib in other cancer types. Glioma H4 cancer cell line, ovarian cancer cell line CAOV-3, and lung cancer cell line NCI-H1975 were treated vehicle control (Ctrl), 4 uM erlotinib, 0.4 mM to 1.6 mM phenformin, 1 mM or 2 mM metformin, or combination for 72 hr. Apoptotic cells were detected with Annexin-V FITC and PI staining.

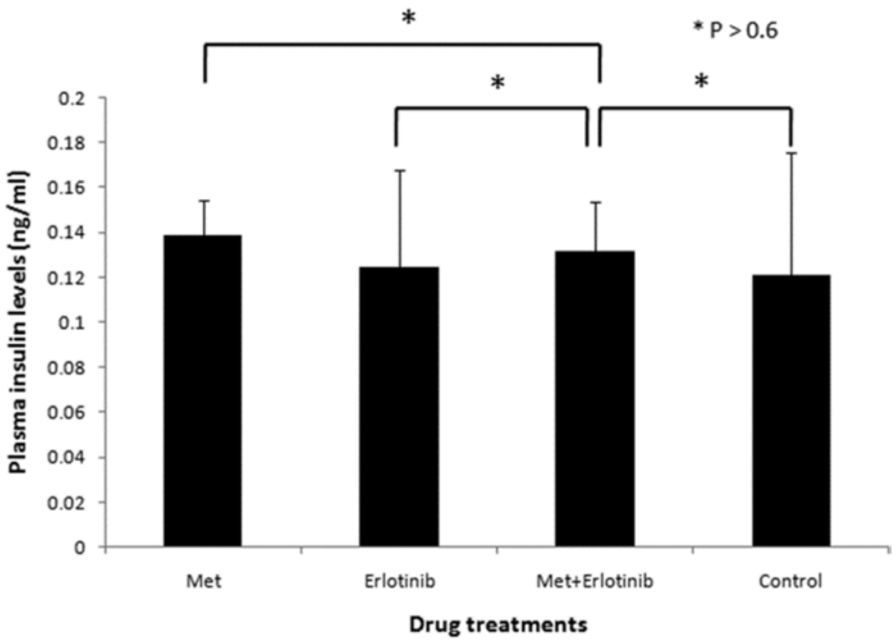

Supplementary Figure S8: Metformin does not alter plasma insulin level in mice. No significant difference in plasma insulin level was observed among the 4 cohorts of mice under different treatments. Plasma was harvested from mice 24 hours after the last dose of metformin and erlotinib was administered. Mouse plasma insulin level was measured using ALPCO Insulin Assay according to manufacturer's instructions.

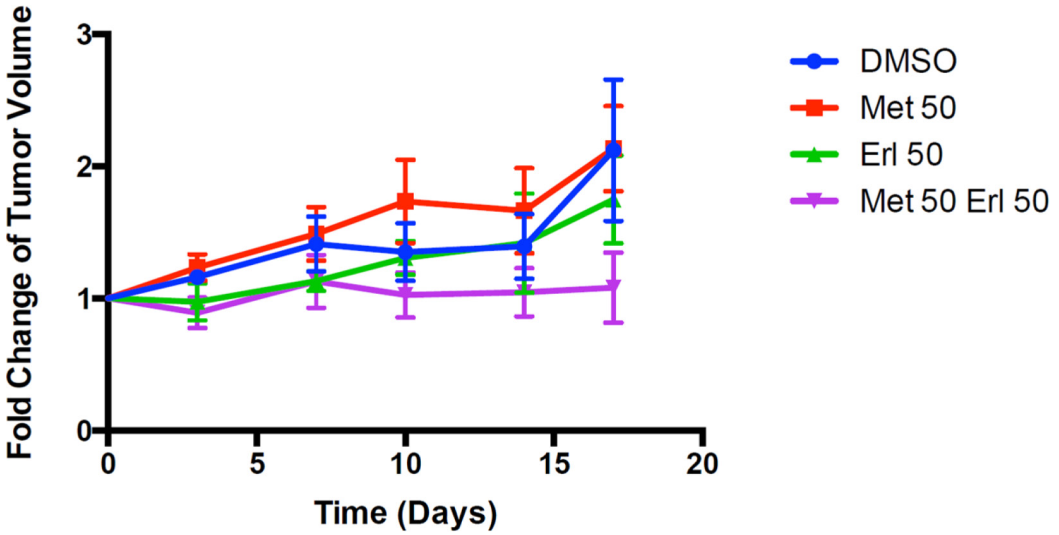

Supplementary Figure S9: Combined erlotinib and metformin inhibit xenografted HCC-70 cells. Two million HCC-70 cells mixed with Matrigel were injected into a posterior mammary fat pad of each mouse and allowed to grow into tumors with size of approximately 100 mm3. The mice were randomized into the indicated arms and treated with metformin alone (50 mg/kg/day), erlotinib alone (50 mg/kg/day), metformin and erlotinib, or vehicle control (DMSO) through daily oral gavage (6 days per week). Tumor size was measured every 3 days. One of two representative experiments is shown (n=4-5 per arm).

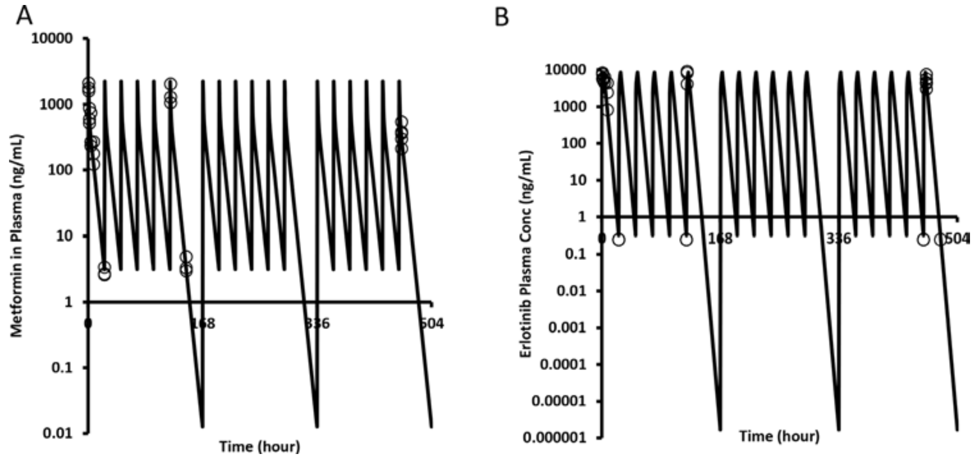

Supplementary Figure S10: Pharmacokinetics study. The mice were treated with metformin (50 mg/kg/day) and erlotinib (50 mg/kg/day) through oral gavage (3 mice per group) on D1. Blood was collected at different time points, 1 hr, 2 hr, 4 hr, 8 hr and 24 hr. Other group mice were continuously treated with metformin (50 mg/kg/day) and erlotinib (50 mg/kg/day) daily. On D6 trough level group was collected, and mice were treated with metformin and erlotinib, and blood was collected 1 hr later. Other mice were treated continuously for 3x6 consecutive days interrupted by one day without treatment. On the last day blood was collected 4 hr after treatment. **(A)** the measured and simulated plasma concentrations of metformin during the combination treatment regimen. **(B)** the measured and simulated plasma concentrations of erlotinib during the combination treatment regimen.

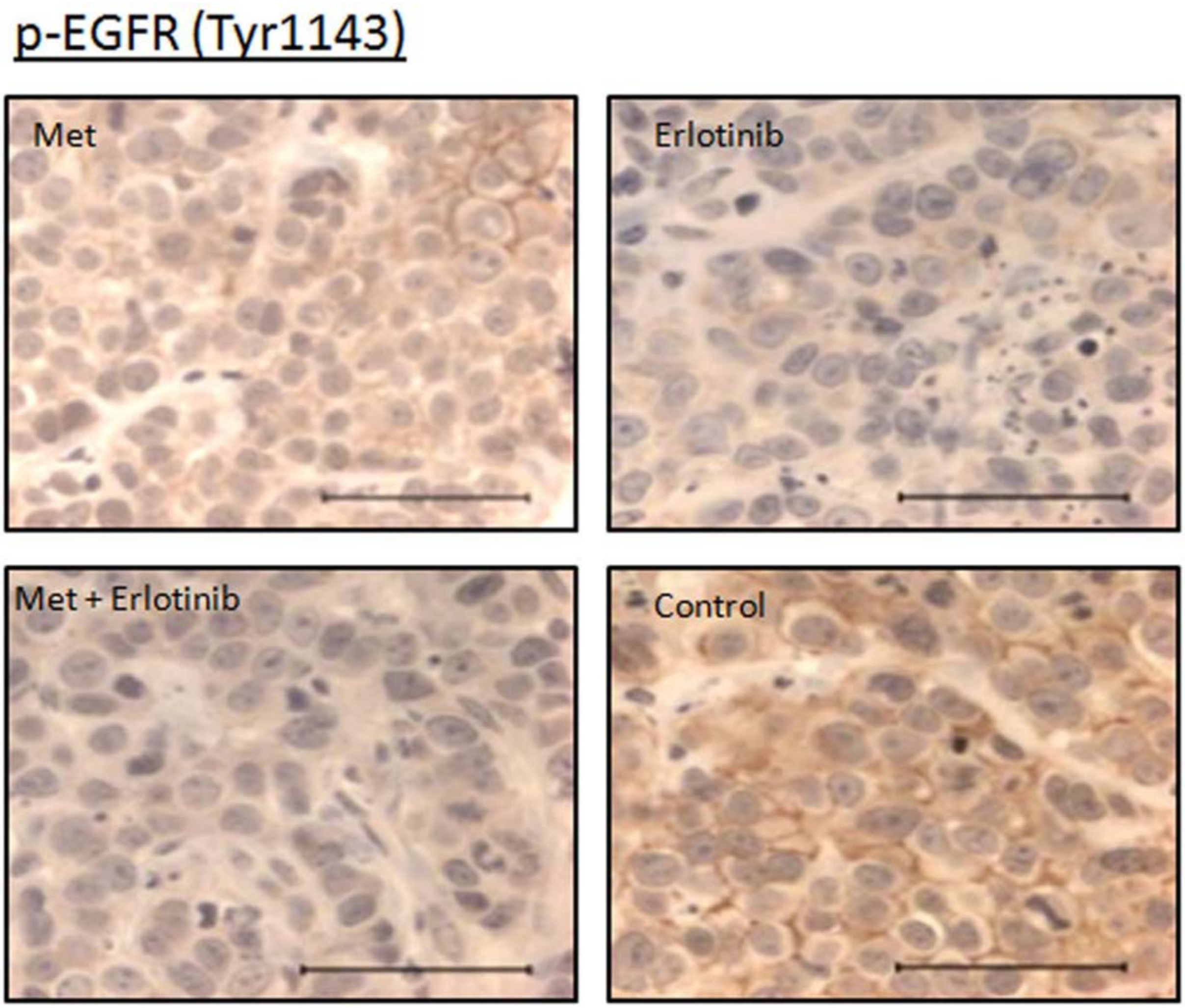

Supplementary Figure S11: Combined treatment of metformin and erlotinib significantly decreased signaling of EGFR in tumor samples. Signaling status of EGFR in tumors excised 24 hours after last drug treatments was assessed by IHC. Length of scale bar equals 50 µM. These are enlarged images from Figure 5D to show the membranous staining more clearly.

Supplementary Table S1: Combined treatment of metformin and BIBW2992 has synergistic effect in MDA-MB-468. MDA-MB-468 cells were seeded at 10,000 cells per well in 48-well plate and subjected to drug treatment of metformin (0.5, 1, 2, 4 mM) and BIBW2992 (0.5 and 1 µM) and their combinations over 6 days. 6 replicates were used per condition. Cell viability was analyzed using crystal violet staining. Combination indices (CI) were determined by using the Compusyn software.

MDA-MB-468

Metaformin(mM)	BIBW 2992 (µM)	Combination Index (CI)
0.5	0.5	0.88
1	0.5	0.67
2	0.5	0.6
4	0.5	0.63
0.5	1	1.04
1	1	0.75
2	1	0.78
4	1	0.53
